# Draft genome assembly of the largemouth bass (Micropterus salmoides)

**DOI:** 10.1186/s12863-025-01318-1

**Published:** 2025-04-16

**Authors:** Tao Zhu, Hongmei Song, Jinxing Du, Caixia Lei, Jing Tian, Chenghui Wang, Chuanju Dong, Shengjie Li

**Affiliations:** 1 Key Laboratory of Tropical and Subtropical Fishery Resources Application and Cultivation, Ministry of Agriculture and Rural Affairs, Pearl River Fisheries Research Institute, Chinese Academy of Fishery Sciences, Guangzhou, 510380 China; 2 College of Fisheries and Life Science, Shanghai Ocean University, Shanghai, 201306 China; 3 College of Fisheries, Henan Normal University, Xinxiang, 453004 China

**Keywords:** Aquaculture, Largemouth bass, Genome assembly, Genome annotation

## Abstract

**Objective:**

Largemouth bass (Micropterus salmoides, LMB) is an important species in aquaculture, and the annual production is rapidly increasing. Genetic and breeding studies related to LMB have promising applications, and a high-quality genome assembly is essential for interpreting genetic and sequencing data. In this study, we sequenced the genome of a male LMB using the PacBio Sequel platform, high-throughput chromosome conformation capture (Hi-C), and paired-end Illumina sequencing. Additionally, Full-length transcript sequencing was performed using isoform sequencing (Iso-Seq). Following the assembly and annotation, a draft assembly for male LMB was obtained.

**Data description:**

This work generated PacBio data of 164.5 Gb, Hi-C data of 113.4 Gb, Illumina data of 54.7 Gb, and Iso-Seq data of 22.8 Gb. The assembly revealed that the LMB genome has a total length of 877.7 Mb, with an N50 of 37.2 Mb, comprising 23 chromosomes and 202 scaffolds. Annotation results indicated that 32.8% of the genome consists of repetitive sequences, containing 23,952 coding genes with an average gene length of 17,328 bp.

## Objective

The Largemouth bass (Micropterus salmoides; LMB), native to North America, is a carnivorous freshwater fish belonging to the Centrarchidae family. Known for its superior meat quality and absence of intermuscular bones, the LMB has been introduced and cultivated worldwide [1]. Since its introduction to China in 1970, the farming area for LMB has been rapidly expanded [2]. Following artificial breeding and domestication, LMB has adapted to aquaculture environments, with an annual output of over 700,000 tons. To promote LMB breeding, extensive genetic and breeding research is currently underway, focusing primarily on genetic diversity [3, 4], whole genome association analysis of growth traits [5, 6], molecular mechanisms of sex determination [7, 8], and tolerance to environmental stress [9, 10], etc. All related research fields require a genome assembly of the LMB. Currently, three genomes of LMB have been assembled [11, 12], which include two female and one male individuals, all from different breeding populations. Here, we sequenced a male LMB from a new population and annotated its gene structure and function, providing further insights and research basis for subsequent LMB genome studies.

## Data description

The sequencing individual was collected from Guangdong Liangshi Aquatic Seed Industry Co., Ltd. (23.19N, 112.78E) and anesthetized with MS- 222. The sex was determined by the sexual gland, and individuals with mature testis were selected for sampling. The sampled tissues include muscle, liver, spleen, kidney, gonad, and intestines. Genomic DNA was extracted from muscle and sequenced on three platforms: paired-end Illumina (PE), PacBio Sequel (PacBio), and Illumina HiSeq X Ten (Hi-C). The three platforms generated PacBio data for 164.5Gb (Table 1, Data file 1, [13]), Hi-C data for 113.4Gb (Table 1, Data file 2, [14]), and Illumina data for 54.7Gb (Table 1, Data file 3, [15]). RNA from various tissues was extracted using Trizol reagent (Invitrogen, CA, USA) and sequenced with PacBio full-length isoform sequencing (ISO-seq, Pacific Biosciences, CA, USA), which generated ISO-seq data for 22.8Gb (Table 1, Data file 4, [16]). Raw Iso-Seq reads were processed using the IsoSeq pipeline to obtain polished consensus sequences. As a result,187,738 consensus sequences with an average length of 2063.07 bp were generated (Table 1, Data file 5, [17]).

**Table 1:** Overview of data files and data sets

Label	Name of data file/data set	File types (file extension)	Data repository and identifier (DOI or accession number)
Data file 1	Raw WGS long reads	Fastq file (.fastq.gz)	NCBI (https://identifiers.org/ncbi/insdc.sra:SRR12489157) [13]
Data file 2	Raw Hi-C reads	Fastq file (.fastq.gz)	NCBI (https://identifiers.org/ncbi/insdc.sra:SRR12605950) [14]
Data file 3	Raw illumina HiSeq reads	Fastq file (.fastq.gz)	NCBI (https://identifiers.org/ncbi/insdc.sra:SRR12443991) [15]
Data file 4	Raw ISO-seq reads	Fastq file (.fastq.gz)	NCBI (https://identifiers.org/ncbi/insdc.sra:SRR31179476) [16]
Data file 5	Full length transcripts consensus sequencing	Fastq file (.fastq.gz)	NCBI (https://identifiers.org/ncbi/insdc.sra:SRR12489156) [17]
Data file 6	Genome survey	Text file (.txt)	Figshare (10.6084/m9.figshare.26391472.v2) [18]
Data file 7	Assembled genome	Fasta file (.fasta)	NCBI (https://identifiers.org/ncbi/insdc.gca:GCA_019677235.1) [19]
Data file 8	BUSCO assessment of the assembly	Text file (.txt)	Figshare (10.6084/m9.figshare.26391472.v2) [18]
Data file 9	Merqury spectra-cn plot	Portable network graphics (.png)	Figshare (10.6084/m9.figshare.26391472.v2) [18]
Data file 10	Genome synteny and collinearity analysis	Portable document format(.pdf)	Figshare (10.6084/m9.figshare.26391472.v2) [18]
Data file 11	Predicted gene	Spreadsheet(.xlsx)	Figshare (10.6084/m9.figshare.26391472.v2) [18]
Data file 12	Predicted genes - nucleotide sequences	Fasta file (.fasta)	Figshare (10.6084/m9.figshare.26391472.v2) [18]
Data file 13	Predicted genes - translated sequences	Fasta file (.fasta)	Figshare (10.6084/m9.figshare.26391472.v2) [18]
Data file 14	Gene annotation using GO, interPro a, KEGG, NR, Swissprot, TrEMBL.	Spreadsheet(.xlsx)	Figshare (10.6084/m9.figshare.26391472.v2) [18]
Data file 15	Predicted ncRNA	Spreadsheet(.xlsx)	Figshare (10.6084/m9.figshare.26391472.v2) [18]
Data file 16	Comparison of assembly and annotation quality	Spreadsheet(.xlsx)	Figshare (10.6084/m9.figshare.26391472.v2) [18]

In the genome survey, we used FastQC (v0.11.8) for quality control and Jellyfish (v2.3.0) (kmer = 17) to estimate the genomic heterozygosity and size [20]. The genome size was determined to be 870.16 Mb, with a heterozygosity of 0.20% and a repetitive sequence proportion of 43.40% (Table 1, Data file 6, [18]). In genome assembly, we used the MECAT2 (v20190314) to assemble PacBio reads [21], used Racon (v1.3.1) and Pilon (v1.22) to correct the base errors [22, 23], Juicer (v1.6) with Hi-C data to generate the interaction maps, and JuiceBox (v1.22) to correct the assembly error [24]. The final assembled genome was 877,669,248 bp in length, consisting of a total of 21 chromosomes, along with 202 long scaffolds, resulting in an N50 of 37.2 Mb (Table 1, Data file 7, [19]). Then, the PacBio reads was mapped to the assembly using the minimap2(v2.17) software [25], showing an alignment rate of 91.48%. To evaluate the quality of the assembly, BUSCO (v4.0.1) and merqury (v1.3) software were used to assess completeness [26, 27]. The BUSCO results indicated that the assembly completeness was 97.8% (Table 1, Data file 8, [18]). The merqury software showed a QV score of 34.91 and a completeness of 97.01%. In the spectra-cn plot, a homozygous peak was found at 53X coverage, suggesting a highly complete and accurate assembly (Table 1, Data file 9, [18]). Compared to another LMB genome GCA_014851395.1, the two genomes shared 820.49 Mb of collinearity blocks, which is over 95.2% of the autosomes (Table 1, Data file 10, [18]).

In repetitive sequence annotation, the tandem repeat was identified by Tandem Repeat Finder (v4.09) software [28], the known repetitive sequence was identified by RepeatMasker (open- 4.0.9), RepeatProteinMask (open- 4.0.9), and Repbase databases, the de novo repeat was identified by the RepeatModeler (open- 1.0.11) and LTR-FINDER (v1.0.5) [29]. Analysis revealed that the genome of LMB contained 43.41% repetitive sequences, among which DNA transposons and SINE accounted for more than 32.8%.

In gene prediction, three methods were used: de novo, homology-based, and transcriptome sequencing-based gene predictions. AUGUSTUS (v3.3) and Genscan were used for de novo prediction [30]; Exonerate (v2.2.0) was employed for homology-based gene prediction, using six species as references. TransDecoder was used for transcriptome sequencing-based gene predictions. MAKER (v3.00) was used to integrate the prediction results of the three methods [31]. As a result, 23,952 non-redundant genes were obtained, with an average gene length of 17,328.32 bp (Table 1, Data file 11, 12 and 13 [18]).

In gene function annotation, BLASTP (v2.6.0+) was employed to align gene sequences to the NR, TrEMBL, InterPro, Swiss-Prot, KEGG, and GO databases [32]. Through this approach, a total of 23,303 genes were successfully annotated (Table 1, Data file 14, [18]). For noncoding RNA prediction, tRNAscan-SE (v1.3.1) was used to identify the tRNA [33], RNAs were identified using the blastn program against related species sequences. miRNAs and snRNAs were identified using Infernal (v1.1.2) software against the Rfam (v14.1) database [34, 35]. In total, 471 miRNA, 2,683 tRNA, 232 rRNA, and 1,200 snRNA were annotated (Table 1, Data file 15, [18]).

## Limitations

The genome sequencing strategies in this study included PE, Hi-C and PacBio. Although a draft genome was assembled, its quality has not significantly improved compared to other assemblies (Table 1, Data file 16, [18]), and the gaps still exist. In the future, a gap-free, telomere-to-telomere genome will be needed.

## Data Availability

The data described in this Data note can be freely accessed on NCBI under Bioproject ID PRJNA656383. The assembled genome is available in GenBank under the accession GCA_019677235.1. The annotation file was available in on Figshare with DOI: 10.6084/m9.figshare.26391472.v2.

## Abbreviations

LMB: Largemouth bass
PE: Paired-end Illumina sequencing
Pacbio: PacBio Sequel sequencing platform
ISO-seq: PacBio full-length isoform sequencing
HI-C: High-throughput chromosome conformation capture
SINE: Short interspersed nuclear elements
GO: Gene Ontology
KEGG: Kyoto Encyclopedia of Genes and Genomes
Nr: Non-redundant
